# What we learned in the development of a third-year medical student curricular project

**DOI:** 10.1007/s40037-021-00648-x

**Published:** 2021-01-27

**Authors:** Maria Syl D de la Cruz, Rashida S. Smith, Alexis E. Silverio, Allison R. Casola, Erin L. Kelly

**Affiliations:** Department of Family & Community Medicine, Sidney Kimmel Medical College at Thomas Jefferson University, Philadelphia, PA USA

**Keywords:** Curriculum development, Undergraduate medical education, Health disparities, Quality improvement

## Abstract

The application of continuous systems improvement in medical education can provide actionable information for curriculum development, improvement, and future planning (as reported by Bowe and Armstrong, Acad Med 92:585–92, 2017). After receiving a medical education grant, we developed a curriculum to teach medical students how to use quality improvement (QI) to address health disparities in vulnerable populations. During the process of developing and implementing this curriculum, we learned several lessons.

One of the major surprises was that our proposed project work took much longer to complete than anticipated. This was mainly because we did not have the right team assembled from the beginning. Specifically, we were missing a team member with evaluation expertise, and therefore we did not devise a systematic process for evaluation and assessment. Without periodic checks or timely assessments built into our curriculum design, we received feedback from students after it was too late to implement changes. We realized that our initial research design had some methodological flaws, which we later rectified.

We encountered additional technical challenges during the curriculum implementation. We struggled with various online learning platforms. Through this, we learned the importance of being knowledgeable upfront about the features of learning platforms and adaptable to changing educational technologies. We also learned our curriculum could and should evolve to meet the needs of our learners and faculty. Moving forward, we realize the benefit of applying a quality improvement process to our curriculum development and implementation, which will help us to continuously transform medical education for future health care needs.

**Supplementary Information:**

The online version of this article (10.1007/s40037-021-00648-x) contains supplementary material, which is available to authorized users.

## The story

To meet the growing needs of the American population, the National Academy of Science includes teaching quality improvement (QI) and population health as core competencies in medical school training [1]. This training can help students learn how to address health disparities (HD) and eliminate gaps in care to achieve health equity [2, 3]. Our department at an urban, private medical school received educational support to develop a population health curriculum—to teach medical students how to use QI methods to improve care for vulnerable populations. The three objectives of the novel, multifaceted curricula were for students to learn about: (1) QI foundational principles, (2) HD in certain communities and vulnerable populations, and (3) to apply QI processes to improve the care of a vulnerable population.

Using a previously developed QI curriculum already implemented in the Family Medicine clerkship, we developed an accompanying HD curriculum. In this concurrent QI curriculum, students completed an Institute for Healthcare Improvement foundational course and a *Plan Do Study Act* video lesson. To inform the development of our HD curriculum, we assessed students’ knowledge, attitudes, and perceptions on community-based medicine, patient safety, and care for vulnerable populations to identify curricular and knowledge gaps. The needs assessment survey revealed that most students neither knew how to identify a community or conduct a needs assessment of that community, nor did they feel comfortable addressing HD in various vulnerable populations, specifically among individuals who were incarcerated, homeless, immigrants/refugees, and veterans (Smith RS, Silverio A, Casola AR, et al. Third-year medical students’ self-perceived knowledge about health disparities and community medicine. 2020 [unpublished]). Clerkship directors and medical education team members decided to use the information gathered from the needs assessment survey to create three interactive, online, self-directed HD modules: (1) *Introduction to Community Health Needs Assessment*, (2) *Health and Homelessness*, and (3) *QI and HD*. In addition to completing the modules, students were required to conduct patient chart reviews and design a QI intervention to improve the process of chronic disease management and include an analysis on how this process would impact a vulnerable population of their choice. Students pitched their QI projects to peers and faculty at the end of the clerkship. Throughout the development and implementation of this curriculum, we experienced our share of successes and difficulties and gained critical insight that could be helpful for other medical educators undertaking similar projects.

## Surprising outcomes

During this project, we encountered a few surprises.

### Gaps in our research team

While we did have a robust, interdisciplinary team, we did not have a team member with explicit evaluation expertise. Without an evaluation expert, we did not include tracking of demographic data or pre- and post-test matching for linking purposes, which we realized was a major limitation of our pilot data. During the implementation phase, there were technical problems with our pre- and post-assessment response collection discovered a few months after implementation via student focus group feedback. Due to a lack of educational design experience, we did not prioritize the need for periodic checks or timely assessments of learner feedback. For example, students were required to complete a web-based assignment of a community health needs assessment on a website that was temporarily unavailable. By the time we realized this, a cohort of students had already completed the curriculum. An emphasis on building in curricular evaluation and feedback components from the beginning could have prevented the overlooking of technical issues.

Additionally, our original team consisted of the principal investigator, two research assistants, and an instructional design member. The assigned instructional design member was skilled in curriculum content, but after a few months, we realized that we really needed assistance with the actual online platform build. When a new educational technology member skilled in web platform design joined our team, the platform build took only a few weeks.

### Technical challenges

We also experienced technical challenges because the QI and HD curricula were developed at different times, using two different platforms. Each curriculum had its own pre- and post-assessments, which used separate survey dissemination platforms. After receiving multiple comments from students about confusing instructions, we troubleshot the technical concerns. We combined the QI and HD curricula onto one learning platform and consolidated the pre-and post-assessments onto a single platform. We updated assignment instructions and added an overview of the project to the clerkship orientation. During a focus group toward the end of our first student cohort, we learned that pre- and post-assessments could be taken more than once. Upon review of our data, we found that we had more pre-assessment responses than the number of students registered for the clerkship. Ultimately, we corrected this setup, but its effect remained a major limitation within our pilot data.

## Lessons learned

1. *Be knowledgeable and flexible with the learning platform you use*

Learning platforms are critical to successful implementation of educational programs. A key technical challenge was the need to consolidate curricula from two learning platforms into one. The QI curriculum was created on a new platform that the university promoted at the time. We chose a different learning management system for our HD curriculum because it provided a grade center and was the university’s main learning platform. Early in the curriculum development process, it is helpful to be familiar with each of the tools and features of various learning management systems so that you select the one that is most compatible with your educational needs [4].

2. *Realize that curriculum implementation can and should evolve to suit the needs of your learners/faculty*

Curriculum development is not a static process. Bowe and Armstrong proposed a systems-based framework in medical education as a way to use continuous QI to not only monitor individual components and facilitate timely corrections but also to drive innovation and prepare for long-term goals and needs [5]. Based on review of student feedback, we modified lesson content to eliminate redundant information in one lesson (QI and HD). Additionally, we modified the curriculum implementation to accommodate our needs. In the beginning, we implemented the curriculum only with students at the main clinical site, to identify issues and challenges before expansion to other sites. We eventually expanded to two additional sites, where students presented their process maps to site directors. Based on the needs of the clerkship schedule, we changed the format to consolidate all the activities into two days—a “QI boot camp.” We combined the curriculum because many of our students work at various affiliate sites. The boot camp model allowed all students to return to the school to have an introductory lecture, have dedicated time to complete online lessons, work on their group chart review and QI projects, and present their work to peers and faculty. Through continuous feedback and evaluation, we are able to revise and enhance the curriculum to meet current and future needs.

3. *Assemble the right team, including an expert on evaluation*

The most important lesson we learned is to assemble the right team from the beginning, including an evaluation expert. Program evaluation is a critical step in curriculum development, as this is needed for ongoing improvement, measuring effectiveness, and assessing the continuous learning of the students [6]. A formal evaluation professional could have helped us with the design of our educational project, selection of evaluation methods and instruments, data collection/analysis, and reporting of results [7]. Continuous assessment of learner feedback is essential for systematic program evaluation [5], which would have allowed us to modify and improve educational components in a timely manner.

Once we had an evaluation member as part of our team, we were able to address procedural and technical flaws. This included using consistent study IDs (preventing multiple completions and allowing the matching of pre-and post-data), changing the release timing of the post-assessment to increase the response rate, implementing periodic checks of our online platform, and adding demographic items to the surveys. Finally, our evaluation expert helped us to assess the quality of our data, suggesting a change to a reflection assignment to obtain robust qualitative data to supplement our quantitative responses.

4. *Gather feedback early and frequently*

We learned the importance of evaluating our implementation processes with continuous student feedback. Because we did not build in an ongoing evaluation method, we found out later that website links were no longer up-to-date and that there was redundant material in educational content, so we were able to eliminate one lesson on QI and HD. Based on student feedback about the clarity of our instructions, we streamlined both the curricula and the assessments onto a single platform and added an overview of the project to the clerkship orientation. Assessment of periodic quantitative and qualitative learner feedback is essential to revise a newly implemented curriculum, troubleshoot problems, and evolve the curriculum [5].

Medical educators can utilize focus groups as a helpful, informal way to gather open-ended feedback from students [8] as an alternative to traditional written course evaluations. We recommend a mid-course focus group to identify opportunities to enhance the curriculum in time to be impactful. Our students greatly appreciated the opportunity to provide feedback in this format, and we were able to rapidly develop consensus about course improvements while also discussing overall course improvements to meet the needs of future learners. We have summarized our major lessons learned within the context of Kern’s 6‑step framework for curriculum development in a table in the Electronic Supplementary Material.

## Moral of the story

While there were challenges to our curriculum rollout, we learned a lot of valuable information about design and implementation. We would emphasize the importance of assembling the right team, as many of the struggles we had could have been avoided if we had had an evaluation expert (in our case, a PhD researcher) and someone familiar with educational electronic platforms working with us from the beginning. We are currently working with our evaluation faculty to ensure we are using the correct processes to collect the most valid data, capture the right outcomes, and develop new research questions and next steps. Moving forward, we realize the benefit of applying a continuous QI process to our curriculum development and implementation. This will help us meet our students’ educational needs, evaluate the curriculum’s impact on the clerkship, and transform medical education for the health care needs of the future.

## Supplementary Information

**Table 1** Summary of lessons learned using Kern’s 6‑step framework for curriculum development (Thomas PA, Kern DE, Hughes MT, et al. Curriculum development for medical education: a six-step approach. Baltimore, MD: Johns Hopkins University Press; 2015)
