# Supplementary material for: What we learned in the development of a third-year medical student curricular project
Source: Perspect Med Educ. 2021 Jan 27;10(3):167–70. doi: 10.1007/s40037-021-00648-x (PMC8187514; doi:10.1007/s40037-021-00648-x)
Supplement: Supplementary file 1 — Table 1 Summary of lessons learned using Kern’s 6‑step framework for curriculum development (Thomas PA, Kern DE, Hughes MT, et al. Curriculum development for medical education: a six-step approach. Baltimore, MD: Johns Hopkins University Press; 2015) [file 40037_2021_648_MOESM1_ESM.docx]

**Table 1** Curriculum development and lessons learned using Kern’s 6-step framework (Thomas PA, Kern DE, Hughes MT, et al. Curriculum development for medical education: a six-step approach. Baltimore, MD: Johns Hopkins University Press; 2015)

| **Kern’s 6-Step Framework** | **Curriculum Development and Lessons Learned** |
| --- | --- |
| 1. Problem identification and general needs assessment | - Recognized need for a medical student curriculum that integrated QI with HD - Performed inventory of existing educational resources and found no prior training targeted toward HD or specific vulnerable populations in the clerkship curriculum |
| 1. Targeted needs assessment | - Designed a survey for third-year family medicine clerkship students to identify knowledge gaps in community-based medicine and care for vulnerable populations - **Lesson Learned #1: Be knowledgeable and flexible with the learning platform you use**   - *Our Challenge:* QI and HD curricula initially created on different learning management systems that we later integrated into one   - *Proposed Solution:* Investigate learning platform options early on, become familiar with each of the tools and features of various learning management systems so that you can identify the best platform for the educational program |
| 1. Goals and objectives | - Based on needs assessment data, defined specific measurable objectives for learners after completion of this curriculum:   1. Identify a community and navigate a community health needs assessment   2. Recognize and discuss HD affecting vulnerable communities and populations   3. Apply targeted QI principles to address HD in vulnerable populations |
| 1. Educational strategies | - Utilized existing QI curriculum   - Institute for Healthcare Improvement 102 course “How to Improve with the Model for Improvement”   - *Plan Do Study Act* video lesson - Created three additional online, interactive, asynchronous HD lessons to complement the above:  1. *Introduction to Community Health Needs Assessment* 2. *Health and Homelessness* 3. *QI and HD* |
| 1. Implementation | **Lesson Learned #2: Realize that curriculum implementation can and should evolve to suit the needs of your learners/ faculty**  *Our Challenge:* During implementation, changes in availability of online resources, the clerkship schedule, and student feedback prompted curriculum revisions.  *Proposed Solution:* Curricular components were modified to meet the educational needs of our learners and faculty by applying an educational QI process to our curriculum development |
| 1. Evaluating the effec-tiveness of the curriculum | **Lesson Learned #3: Assemble the right team, including an expert on evaluation**  *Our Challenge:* There were procedural and technical flaws in our pilot data collection because we did not involve an evaluation expert at the beginning of our process    *Proposed Solution:* Including an evaluation expert at the beginning (to assist with design, implementation, data collection, and assessment) would have mitigated a number of the issues noted above.  **Lesson Learned #4: Gather feedback early and frequently**  *Our Challenge:* We did not include ongoing evaluation methods in our curriculum design and implementation.  *Proposed Solution:* Build in formal processes for timely and continuous feedback, which can be used to tailor the curriculum throughout implementation |
